# Cognitive Dimensions of Predator Responses to Imperfect Mimicry

**DOI:** 10.1371/journal.pbio.0050339

**Published:** 2007-12-27

**Authors:** Lars Chittka, Daniel Osorio

## Abstract

How palatable animals manage to enjoy increased protection from predators by imperfectly mimicking well-defended animals, like wasps, even though their resemblance appears slight to the human eye has long puzzled evolutionary biologists. Could predator cognition explain the mimics' success?

Many palatable animals, for example hoverflies, deter predators by mimicking well-defended insects such as wasps. However, for human observers, these flies often seem to be little better than caricatures of wasps—their visual appearance and behaviour are easily distinguishable from those which they are attempting to mimic. This imperfect mimicry baffles evolutionary biologists, because one might expect natural selection to do a more thorough job. Here we discuss two types of cognitive processes that might explain why distinguishable mimics could enjoy increased protection from predation. **Speed–accuracy tradeoffs** in predator decision making might give imperfect mimics sufficient time to escape, and predators under time constraint might avoid time-consuming discriminations between well-defended models and inaccurate edible mimics and instead adopt a “safety first” policy of avoiding insects with similar appearance. **Categorisation** of prey types by predators could mean that wholly dissimilar mimics may be protected, provided they share some common property with noxious prey. If predators use experience with multiple prey types to learn rules rather than just memorising the appearance of individual prey types, it follows that different individual predators should form different categories, each including separate types of novel prey. Experimental studies to test these hypotheses should be straightforward, because we can use the relatively simple signals (e.g., striped patterns) with which prey manipulate predator behaviour as tools for investigating cognitive processes that underlie decision making and object recognition in animals' daily lives.

## Introduction

**Figure 1:**
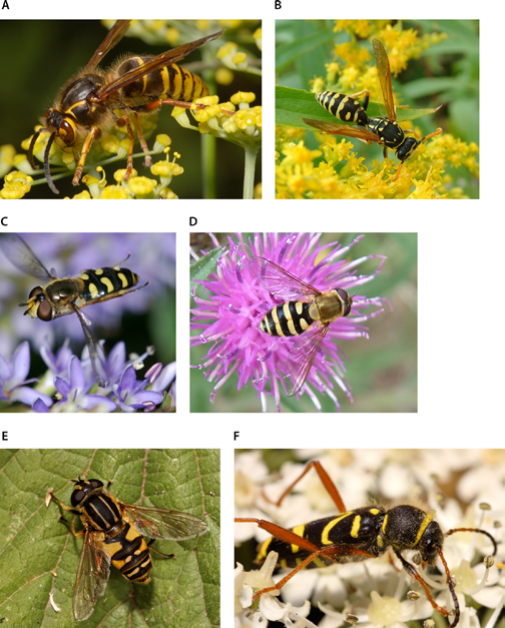
Two Wasp Species and Four Less-Than-Perfect and Palatable Mimics (A) Dolichovespula media; (B) Polistes spec.; (C) Eupeodes spec.; (D) Syrphus spec; (E) Helophilus pendulus; (F) Clytus arietes (all species European). Note that species C–F do not closely resemble any wasp species. The three hoverfly species differ in wing and body shape, antennal length, flight behaviour, and striping pattern from European wasps. One fly species (E) even has longitudinal stripes, which wasps typically don't. The harmless wasp beetle does not normally display wings, and its legs do not resemble those of any wasps. (Image Credit: (A, C, E, and F) by Rob Knell; (B and D) by Tom Ings)

Mimicry—the phenomenon where organisms converge in appearance on one another, often to warn or deceive predators—provides examples of adaptive evolution so striking that they should convince even staunch sceptics of the principles of evolution. Perfectly harmless caterpillars look like venomous snakes, while angler fish display lures that resemble small fish. In many other cases, however, the match between the mimic and its model is almost disappointingly sloppy. Take many of the familiar hoverflies: their yellow and black stripes might resemble a stinging wasp to an inexperienced observer—but the body shape, flight behaviour, and colour pattern of many species easily identify them as defenceless flies (Figure 1). Yet, the strategy works! The flies' colouration pattern provides protection that they would not enjoy if they were, say, plain brown [1]. The suspicion that such imperfect mimics might not in fact be mimics at all was refuted already in 1935, when Mostler [2] demonstrated that inexperienced, lab-reared birds of several species would not only enthusiastically attack bumblebees, honeybees, wasps, and their mimics, but the birds would learn to reject these and also avoid relatively crude mimics if they were offered after an encounter with a wasp. The syrphids thus engage in so called Batesian (deceptive) mimicry, where a palatable animal mimics the display of a noxious model. Imperfect mimics also occur in vertebrate colour displays, for example in some North American snakes [3].

Several evolutionary scenarios have been proposed that might explain such imperfect mimicry. One suggestion that is relatively difficult to test is that mimics have not had sufficient time to converge fully on the model (see [1] for a critique). Another possibility is that models and mimics are engaged in an evolutionary arms race, where the model is under pressure to evolve away from the mimic [4]. This is because predators are more likely to attack noxious prey after encounters with individuals of similar palatable species ([2], but see also [5]). Some researchers have related the degree of similarity in mimicry systems to the relative frequencies of models and mimics [3,5], while others pointed out that there are conflicting demands on animals' colour patterns, resulting in compromises between signalling strategies and, for example, constraints of thermoregulation [6]. The number of controversial views aired in high-profile journals indicates that biologists are clearly intrigued by the problem, but good experimental evidence for many scenarios still needs to be collected. Our view is that we cannot quantify the evolutionary pressures on animal colour patterns without considering what is known about predators' cognitive abilities. In some cases, we suggest that the peculiarities of predator “receiver psychology” might result in the full protection of mimics, even if these only partially resemble their models and both are distinguishable by predators—resulting in a lack of selective pressure to increase the similarity between a mimic and its model(s).

A simple psychological explanation for predator responses to poor mimics could be that predators innately avoid any stripy pattern. Such innate biases do exist [7], but typically they are weak and can easily be overwritten by learning [1,2,4]. Therefore, current explanations of imperfect mimicry refer to predators' individual experience with unpleasant mimics, and responses to mimics that are guided by such experience. Previous explanations of imperfect mimicry include the following: (a) the possibility that differences in visual systems between humans and insectivores (typically avian predators) might mean that what constitutes a poor match for human observers might in fact be perfect mimicry for some predators [8]; (b) in the presence of multiple aposematic models, mimics attempt to find a compromise by appearing intermediate to all of them [1,9]; and (c) generalisation of predators to distinguishable but similar prey might give sufficient protection for poor mimics [4,10–12]. These explanations are not mutually exclusive, and empirical evidence is scant [1]. However, the predator learning processes that have been discussed in the context of mimicry are essentially Pavlovian, in that they invoke only simple processes of information storage, generalisation, and forgetting [13], and thus do not fully capture the range of cognitive abilities that predators might use. Cognition can be defined as the ability to use internal representations of information acquired in separate events, and to combine these to generate novel information and apply it in an adaptive manner [14] —a classic example is the cognitive map, where subjects integrate information from separately travelled paths to calculate new routes [15]. In contemporary animal behaviour, there is a general fascination with probing the level of cognitive complexity that animals can achieve. Not applying the fruits of this research to animals' natural lives would be a major oversight—we cannot continue to regard animals as simple “conditioned reflex machines” if we are to understand the complexity of interactions between signallers and receivers, especially where receivers might combine experience with multiple signallers to form rules for adaptive behaviour. Here we discuss two cognitive abilities that allow predators to make effective decisions about whether or not to attack, while maintaining a low level of risk of confusing a nutritious mimic with its noxious model. These processes may be exploited by imperfect mimics.

## Speed–Accuracy Tradeoffs in Animal Decision Making?

Everyday experience shows that difficult perceptual tasks require more time than easy tasks do. If time is limited for difficult judgments, one is more likely to make mistakes. Consider a hypothetical football match where one team wears green and the other turquoise. The two colours are easily distinguished, but as players continuously change position and mingle with one another, the time for classifying them as members of one or the other team will be limited. The result is confusion of green and turquoise that will make the match substantially less enjoyable. Conversely, when it is essential to avoid mistakes, more time is needed. A mushroom collector has to make triply sure not to mistake a death cap (Amanita phalloides) for the similar and edible false death cap (Amanita citrina). If, after extensive inspection, there is any uncertainty, a false alarm is obviously preferable to a fatal error. Understanding such speed–accuracy tradeoffs is an essential part of contemporary decision theory [16].

In bees and mice, just as in humans, sensory discrimination typically improves with the time allowed for a decision, and difficult discrimination tasks require more time to be solved with high accuracy [16–19]. Such speed–accuracy tradeoffs result from the need to sample information over time in noisy conditions, so that evidence for competing options accumulates until a decision threshold is reached [17,20–22]. Thus, although the mechanistic causes of speed–accuracy tradeoffs might sometimes lie in low-level sensory processes, devising strategies that take into account such mechanistic limitations requires error awareness and attention, i.e., cognitive processes. Such tradeoffs should be of fundamental importance to animal decision making in the economy of nature, but their relevance in the natural lives of animals has only recently been considered [18,23–25]. There are obvious implications for predators when similar mimics must be discriminated from noxious models, especially in time-constrained situations, such as scramble competition or when the prey might escape. Data on speed-accuracy tradeoffs for avian predators are still outstanding, but we suggest possible avenues of future research below.

## Testing the Role of Speed–Accuracy Tradeoffs in Predators Judging Inaccurate Mimics

An appropriate test of the interaction between choice time and precision of choice needs to involve prey items that are only briefly on display, or moving, rather than stationary, and with no time limitations. Because there are ethical concerns with experimental designs where birds might be stung by insects, live prey cannot be used; instead penalties might consist of food rendered unpalatable with bitter quinine solution [18]. It will be essential to vary the display time or movement speed, as well as the number (and perhaps direction of movement) of palatable and unpalatable prey, to mimic the crowded conditions that predators might encounter in nature. Both sequential and simultaneous choice should be tested.

It will first be necessary to quantify the speed–accuracy trade-off depending on the similarity between unpalatable models and palatable mimics. Emphasis can be placed either on accuracy (by varying the severity of punishment for errors) or speed (by limiting the time available for an attack). Once such baseline data are established, two predictions are especially worth testing. One is that if discrimination between a model and a mimic costs appreciably more time, even relatively inaccurate mimics might gain time to escape [26]. Consider your own response to a yellow-and-black hoverfly approaching you on a summer day: the first reaction might be that you are temporarily alarmed, even though close (but time-costly) inspection might identify it as harmless. The second prediction is that a predator, under time constraint, will avoid time-costly discriminations between defended models and inaccurate edible mimics, and instead adopt a “safety first” policy of avoiding all insects with similar appearance. This could be tested by offering three types of prey that vary in colour and palatability: for example, A: red, unpalatable—the aposematic model; B: red-orange—a “mimic” similar to A, but palatable; and F: blue, palatable but distinct from A. An optimal forager should choose B and F, but there is of course the risk of errors (“confusing” A with B). Thus, in a situation when time is limited, predators should go for safe option F. However, this would involve false alarm errors, avoiding the profitable B, and halving the intake rate. These experiments should identify the range of similarity in which speed–accuracy tradeoffs mean that inaccurate mimics might not only enjoy improved protection from predators relative to palatable insects without aposematic colouration, but also, critically, that a further increase in similarity to the model might confer no further fitness benefits.

## Categorisation of Food Types by Animals

Categorisation allows us to classify stimuli in meaningful way (e.g., as dogs, cats, chairs, tables, etc.) and independently of their individual shape and colour. Note that categorisation differs from generalisation. Generalisation allows animals to attribute common properties to distinguishable objects; however, the level of similarity can vary in a continuous fashion, as when one sees a greater similarity of yellow to orange than to red, and likewise of yellow to lime than to green. On a continuous sensory dimension, such as the visible spectrum, the extent of generalisation from a given stimulus value (e.g., wavelength of light) typically has a Gaussian or exponentially shaped function centred on that value [10,27,28]. One might expect the extent of generalisation to be related to sensory discrimination thresholds, and hence to be related to the speed–accuracy trade-off. By comparison, categories have definite boundaries—an object is either a member of a category or not—and they can include diverse or entirely dissimilar items, such as dogs or fruit. However, a category has some defining feature that is common to all its members. Categorisation may also be understood as a strategy for being economic with memory—by extracting the cues that define a class of objects, rather than just a single object, an animal might circumvent having to memorise the appearance of dozens of salient objects [29].

A predator without categorisation might make almost inconceivably inappropriate judgments: consider an animal that, after being stung in the tongue by a black-and-red bumblebee, treats a black/yellow/white striped bumblebee as potentially palatable. Hence, categorisation is adaptive, but there is a risk of “false alarm” errors, where palatable mimics (even if they bear no direct similarity to aposematic prey) fall within an avoided category. Pigeons and chicks have been shown to be able to form categories [28,29]; for example, Cerella [30] made a good case that pigeons recognise oak leaves as a natural category. In particular, after learning a single oak leaf shape, they did not discriminate between a wide range of oak leaves, but reliably distinguished oaks from leaves of other species. As with tree leaves, aposematic insects such as wasps, bumblebees, and shield-bugs (Pentatomidae) have a characteristic shape that birds might recognise as natural kinds; alternatively, they might classify patterns according to whether or not they contain more than one colour (independently of the particular combinations of colours).

## Testing the Role of Prey Categorisation in Insectivores

Rather than just associating one colour pattern with an unpleasant experience, do predators learn the rules for classifying patterns, such as those that are displayed by toxic insects, to predict whether an unfamiliar species of insect is safe to eat? In human education, a successful strategy is first to learn the rules, then the exceptions. If birds first learn the basic principles of warning colouration, then even poor mimics might enjoy protection, especially when predators have to make rapid judgements (see above). For example, after a predator has had unpleasant encounters with two distinct bumblebee species, it might categorise by prey shape and not colour, and subsequently avoid all bumblebees irrespective of colour banding pattern.

**Figure 2:**
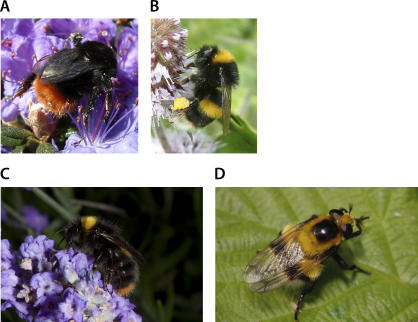
Colour Coats of European Bumblebees and a Stingless Mimic (A) Bombus lapidarius; (B) B. terrestris; (C) B. pratorum; and (D) the fly Volucella bombylans. Note that (B) and (D) are considered part of the same mimicry ring [31], even though they are clearly distinct. But, a predator categorising by shape might respond equally to both, as to the highly distinct B. lapidarius (A), and the individual of the fly V. bombylans (D), which looks like no particular central European bumblebee species, but captures the overall essence of a bumblebee-like appearance (body shape, hair coat, and some form of stripes). (Image credit: (A and B) by Tom Ings; (C) by Mike Edwards; and (D) by Rob Knell)

An especially interesting question concerns the way in which animals establish categories after learning about a number of distinct stimuli that share common properties. It is widely thought that groups of similar but discriminable prey species form so-called “mimicry rings” [1,4,31] (Figure 2). Often, the participant species engage in Müllerian (“honest”) mimicry, where multiple, defended species converge on one another in appearance, so that individuals of one species can profit from what a predator has experienced in an encounter with a member of a different species [4]. There is experimental evidence that birds can establish well-defined colour categories from multiple examples [27]. In nature, after being exposed to two or more different prey (e.g., wasp) species that differ in shape and colour but share a high-contrast stripe pattern, birds might categorise by pattern and irrespective of shape, therefore including some imperfect mimics (e.g., hoverflies) despite their difference in body shape. These questions should be straightforward to address experimentally by using sequential exposure to different prey. Understanding how avian predators classify the range of patterns that are displayed by hymenopterans and their mimics, depending on individual experience, and the cues that they extract to form categories will give valuable insights into the evolution of mimicry and also provide a naturalistic context in which to address wider questions about the cognitive processes that underlie object recognition in nonhuman species [32]. The differences between responses following training to single and multiple examples will give important information about the natural history of mimicry rings and the underlying cognitive processes. An important (and untested) prediction is that if predators use experience with multiple prey types to learn rules rather than just memorising the appearance of individual prey types, it follows that different individual predators should form different categories, each including separate types of novel prey—depending on individual experience.

## Conclusion

Mimicry is one of the most venerable and at the same time most dynamic areas in whole-organism biology. Recent developments in animal cognition now make it possible to understand not only how animals perceive mimicry systems [8,33,34], but also how they store information about such systems, how such information consolidates and changes with experience and with time [35–37], and how animals might extract the general rules by which animal colouration and palatability are linked. Incorporating realistic time constraints into experiment designs, and the visual information-processing speed of predators, should help identify the conditions under which the cognitive processes of predators will sometimes generate space for inaccurate mimics to live.
